# Stronger coupling of emotional instability with reward processing in borderline personality disorder is predicted by schema modes

**DOI:** 10.1017/S0033291723000193

**Published:** 2023-10

**Authors:** Gábor Csukly, Kinga Farkas, Tímea Fodor, Zsolt Unoka, Bertalan Polner

**Affiliations:** 1 Department of Psychiatry and Psychotherapy, Semmelweis University, Balassa street 6, Budapest 1083, Hungary; 2 Department of Cognitive Science, Budapest University of Technology and Economics, Egry József street, Building T, Floor 5, Budapest 1111, Hungary; 3 Institute of Psychology, ELTE, Eötvös Loránd University, Izabella utca 46, Budapest 1064, Hungary

**Keywords:** Computational psychiatry, decision-making, hierarchical Bayesian analysis, impulsivity, mood instability, personality disorders, reward prediction error, reward processing, risky decision, schema modes

## Abstract

**Background:**

Mood instability and risk-taking are hallmarks of borderline personality disorder (BPD). Schema modes are combinations of self-reflective evaluations, negative emotional states, and destructive coping strategies common in BPD. When activated, they can push patients with BPD into emotional turmoil and a dissociative state of mind. Our knowledge of the underlying neurocognitive mechanisms driving these changes is incomplete. We hypothesized that in patients with BPD, affective instability is more influenced by reward expectation, outcomes, and reward prediction errors (RPEs) during risky decision-making than in healthy controls. Additionally, we expected that these alterations would be related to schema modes.

**Methods:**

Thirty-two patients with BPD and thirty-one healthy controls were recruited. We used an established behavioral paradigm to measure mood fluctuations during risky decision-making. The impact of expectations and RPEs on momentary mood was quantified by a computational model, and its parameters were estimated with hierarchical Bayesian analysis. Model parameters were compared using High-Density Intervals.

**Results:**

We found that model parameters capturing the influence of RPE and Certain Rewards on mood were significantly higher in patients with BPD than in controls. These model parameters correlated significantly with schema modes, but not with depression severity.

**Conclusions:**

BPD is coupled with altered associations between mood fluctuation and reward processing under uncertainty. Our findings seem to be BPD-specific, as they stand in contrast with the correlates of depressive symptoms. Future studies should establish the clinical utility of these alterations, such as predicting or assessing therapeutic response in BPD.

## Introduction

Mood Instability and risk taking are hallmark features of borderline personality disorder (BPD). They greatly contribute to the distress, functional impairment, disease burden, and socio-economic costs associated with the condition. BPD is present in roughly 1% of the general population, around 10–12% of psychiatric outpatients, and 20–22% of inpatients (Ellison, Rosenstein, Morgan, & Zimmerman, 2018). Critical features of BPD are pervasive instability of interpersonal relationships, self-image, identity, mood, and emotions. Patients with BPD often demonstrate dissociative states and impulsivity that lead to non-suicidal self-harming behavior, frequent suicide attempts, and a high level of suicidal lethality (American Psychiatric Association, 2013; Lieb, Zanarini, Schmahl, Linehan, & Bohus, 2004). Suicidal ideation and behavior in BPD are strongly predicted by pervasive instability of affective states (Rizk et al., 2019), plus high levels of intense negative mood and mood variability (Links et al., 2007). Mood variability appears to be related to trait impulsivity, which in turn is strongly related to schema modes in BPD (Boog et al., 2021).

Schema modes are combinations of the activated self-reflective evaluations, negative or positive emotional states, and coping strategies, being the momentary reflections of the individual's emotional, cognitive, and behavioral state. When maladaptive schema modes are active, the person is in a dissociative state of mind. Schema modes are organized into four categories: child modes, coping modes, parent modes, and the Healthy Adult mode. Modes can be adaptive (the Healthy Adult and the Happy Child mode) or maladaptive (every other mode) (for a short description of schema modes, see Table 1) (Salgó, Bajzát, & Unoka, 2021; Young, Klosko, & Weishaar, 2006).

**Table 1.** Schema modes

Categories	Schema modes
Healthy mode	Healthy Adult is an adaptive mode that reflects on the present states of mind, evaluates the situation in the context of personal history, emotional needs of the person and the others, and solves problems.
Child modes	Happy Child is an adaptive mode, which is experiencing positive feelings because core emotional needs are currently met.
	The Vulnerable Child is characterized by experiencing negative feelings as a consequence of unmet core emotional needs.
	The Impulsive Child is characterized by acting on impulses in an uncontrolled manner without regarding the consequences and difficulty delaying gratification.
	The Angry Child is characterized by intense anger because their core emotional needs are not being met.
	The Undisciplined Child is characterized by the inability to finish routine or boring tasks.
	The Enraged Child is characterized by the feeling of anger to destroy the aggressor.
Dysfunctional coping modes	Compliant Surrenderer is characterized by giving up their own needs in a submissive way to avoid rejection or conflict.
	Detached Protector is characterized by avoiding the pain of unmet core emotional needs by dissociative means like emotional detachment and disconnection from others.
	Detached Self-Soother is characterized by engaging in activities that soothe, stimulate or distract them from feeling: drug abuse, addictive behavior, overeating, promiscuous sex, fantasizing, and others.
	Self-Aggrandizer is characterized by inappropriately entitled, grandiose, competitive, and abusive behavior.
	Bully and Attack is characterized by emotionally, verbally, physically, and sexually abusive acts that are delivered in a controlled and strategic way.
Parent modes	Punitive Parent is the internalized voice of the parent or other influential figures, criticizing and punishing the patient.
	Demanding Parent is the internalized voice of the parent or other influential figures, requiring excessively high standards and perfectionism.

The self-disturbances and impaired interpersonal functioning captured by maladaptive schema modes are core features of personality disorders (Bach & Bernstein, 2019). Thus, high levels of all maladaptive schema modes can be interpreted as a sign of general personality pathology and BPD might be a prototypical example of this dimension. Bifactor modeling of personality disorder criteria data yielded a general personality pathology factor and personality-disorder specific factors (Sharp et al., 2015). While BPD was only related to the general factors, other personality disorders were associated with the general and specific factors as well. Thus, although maladaptive schema modes are the most prevalent in BPD, they are also a transdiagnostic dimensional feature that is related to personality pathology. Abrupt switches of active schema modes in BPD can cause pervasive instability of mental states. Schema modes are measured using retrospective information obtained through a self-report questionnaire [in our case, Schema Mode Inventory (Lobbestael, Vreeswijk, Spinhoven, Schouten, & Arntz, 2010; Young et al., 2007)]. There is a need to go deeper and to catch the immediate consequences of decision-making in a risky context on the mood and its associations with schema modes measured by self-report retrospective schema mode inventory. Adaptive schema modes are a prerequisite of efficient emotion regulation and impulse control (Bach & Bernstein, 2019). In the case of low level of Healthy Adult mode and high level of maladaptive schema modes, a negative feedback in a decision-making situation triggers maladaptive modes and negative mood. Therefore, we argue that they predict how moods will fluctuate in an uncertain decision-making situation where one is at the mercy of luck.

In the present study, we adopted the behavioral task by Rutledge, Skandali, Dayan, and Dolan (2014) to investigate the immediate consequences of reward expectations and outcomes on mood. The main advantage of this approach is that it measures the impact of events on mood changes by putting the subjects in a game-like environment that models decisions and their desirable or adverse consequences. This provides a way to evaluate the impact of events on mood with greater ecological validity as compared to traditional single-assessment self-report questionnaires and interviews.

There are several models on the mechanisms by which an outcome affects mood. Utility-based models assume that agents thrive for the choice with the objectively achievable maximum gain (predicted utility), which they also expect to come with the objectively possible maximum positive change in mood (experienced utility) (Berridge & Aldridge, 2008). An alternative computational model of subjective well-being, however, predicts that a decision's effect on mood will be the result of the expected reward plus its divergence from the realized reward (reward prediction error; RPE), or in other words: how well things are going in comparison to initial expectations. According to the results of Rutledge *et al*. (2014, 2017), this model explains mood changes in the probabilistic reward task better than the utility-based ones. In this relation, mood serves an adaptive role in decision making by modulating the agent's expectations in accordance with experience acquired during previous instances of reinforcement learning. This can increase learning efficiency since the accumulated effects of a situation's individual factors do not have to be accounted for individually (Eldar, Rutledge, Dolan, & Niv, 2016).

An advantage of such a computational approach to subjective well-being is its capability to make the effect of RPE (i.e. outcome – expected reward) on mood quantifiable through model parameters. It also utilizes the method of experience sampling, which involves recording the subject's mood on multiple occasions during an experiment in real-time (Rutledge et al., 2014). Measuring the event-induced fluctuation in mood in a laboratory environment this way is advantageous over other emotion-induction techniques because of its higher ecological validity (Eldar et al., 2016).

While increased risk-taking behavior is a well-replicated finding in BPD (Bohus et al., 2021), to date, no study has evaluated the effect of expected outcomes and the mismatch between expected and realized results on mood in a probabilistic reward task. Despite strong theoretical arguments for affective influences on decision making by Lerner, Li, Valdesolo, and Kassam (2015), who claimed that predicted outcomes of present decisions influence present or future emotions, to our best knowledge, no study has investigated the effect of expected *v.* realized outcomes on emotional states in BPD.

We argue that the paradigm developed by Rutledge et al., models the effect of real-life events and decisions on mood. We propose that the well-documented affective instability in BPD (Santangelo et al., 2014) may stem from enhanced sensitivity to outcomes during risky decision making. Therefore, we expected higher weights in patients with BPD compared to healthy controls. Furthermore, in line with recent dimensional approaches to mental disorders and well-being (Borsboom et al., 2016; Cuthbert, 2020; Kotov et al., 2021; Michelini, Palumbo, DeYoung, Latzman, & Kotov, 2021; Sanislow, 2020), we hypothesized that these weights would show positive correlations with maladaptive schema modes (such as the ‘impulsive child’) that are typically high in BPD.

## Methods

### Participants

Seventy-five participants were recruited (Table 2). Nine subjects were excluded for not indicating a significant (>5%, a control experiment showed that this was the noisy level of our mood measurement) change of mood during the investigation. We also lost the data of three participants due to technical reasons. Finally, data were analyzed from 32 participants with BPD (5 males, mean age 28.4 years) and 31 healthy controls (9 males, mean age 30.4 years). Study groups did not differ significantly in terms of sex, age, and education level. The main demographic and clinical characteristics are shown in Table 2.

**Table 2.** Demographics and clinical characteristics

		Borderline (*N* = 32)			Healthy control (*N* = 31)			Statistics	*p*
		Mean (s.d.)	Min	Max	Mean (s.d.)	Min	Max	Mann–Whitney *U*	
Age	(years)	28.4 (8.9)	18	50	30.4 (9.9)	19	53	*W* = 562	0.367
Illness duration	(years)	7.6 (7.4)	0	24	–	–	–	–	–
Medication	CPZ equivalent	104.7(199.7)	0	1000	–	–	–	–	–
	DZ equivalent	5.4(9.3)	0	30	–	–	–	–	–
		*N*	%		*N*	%		χ^2^	*p*
Sex	M/F	5/27	(15.6%/84.4%)		9/22	(29%/71%)		1.638	0.201
Education	Primary/Secondary/Higher	2/20/10	(6.2%/62.5%/31.3%)		1/13/17	(3.2%/41.9%/54.9%)		3.618	0.164
Smoking	Never/Sometimes/Regular	13/6/13	(40.6%/18.8% /40.6%)		24/5/2	(77.4%/16.1%/6.5%)		11.415	0.003*
Alcohol	Never/Sometimes/Regular	7/19/6	(21.9%/59.4%/18.7%)		24/5/2	(77.4%/16.1%/6.5%)		2.9	0.235
Drug	Never/Tried before/Regular	14/17/1	(43.8% /53.1%/3.1%)		20/11/0	(64.5%/35.5%/0%)		3.33	0.189
Gambling	Never/Tried before/Regular	17/14/1	(53.1%/43.8% /3.1%)		17/14/0	(54.8%/ 45.2%/0%)		0.984	0.611
Treatment	First/Chronic	10/22	(31.2%/68.8%)		–	–		–	–
	Out-/Inpatient/Psychotherapy ward	3/2/27	(9.4%/6.2%/84.4%)		–	–		–	–
Medication type	AP/BZD/AD/MS	13/11/11/18	(41.9%/35.5%/45.5%/58.1%)		–	–		–	–

s.d., Standard Deviation; CPZ, Chlorpromazine; DZ, Diazepam; M, Male; F, Female; AP, Antipsychotic; BZD, Benzodiazepine; AD, Antidepressant; MS, Mood stabilizer.

Inclusion criteria were no history of any central nervous system disease or mental retardation for both study groups. For healthy controls, no history of any psychiatric illness, less than five positive answers on the SCID-II Personality Questionnaire, BPD module (Ryder, Costa, & Bagby, 2008), and global severity index of < 50 on the Symptom Checklist-90-R (Derogatis, Lipman, & Covi, 1973), were additional inclusion criteria to exclude subjects with risk for psychiatric disorders [the Hungarian version of the SCL-90-R: (Unoka et al., 2004)].

Patients were recruited at the Department of Psychiatry and Psychotherapy of Semmelweis University, Budapest, Hungary. The majority of the patients participated in inpatient group psychotherapy treatment (*n* = 27), while the remaining participants were recruited from other inpatient (*n* = 2) or outpatient units (*n* = 3) of the department. All patients met the criteria for BPD based on the DSM-5 (American Psychiatric Association, 2013). At the time of the experiment, some of the patients took antipsychotic (*n* = 13), benzodiazepine (*n* = 11), mood stabilizer (*n* = 11), or antidepressant (*n* = 18) medication. Comorbidity data were collected from electronic health records: seven participants had comorbid depression, five had anxiety disorders, and two had bipolar disorder (euthymic phase at the time of examination), three had eating disorder, one had OCD and one had PTSD. These data correspond to previous studies reporting high comorbidity rates among subjects with BPD (Tomko, Trull, Wood, & Sher, 2014). Participants from the psychotherapeutic program were screened for other personality disorders by SCID-II interview. In addition to BPD, four participants had avoidant, two dependent, eight obsessive-compulsive, seven paranoid, one schizoid, one schizotypal, one histrionic and five narcissistic personality disorder. This finding is in line with previous studies showing high rate of comorbid personality disorders in patients with BPD (Zanarini et al., 1998, 2004).

The study was conducted in accordance with the Declaration of Helsinki. It was approved by the Semmelweis University Regional and Institutional Committee of Science and Research Ethics, Budapest, Hungary (SE RKEB No. 40/2019). Participants gave written informed consent before taking part in the study.

### Experimental paradigm and the computational model of mood fluctuations

**Fig. 1.**
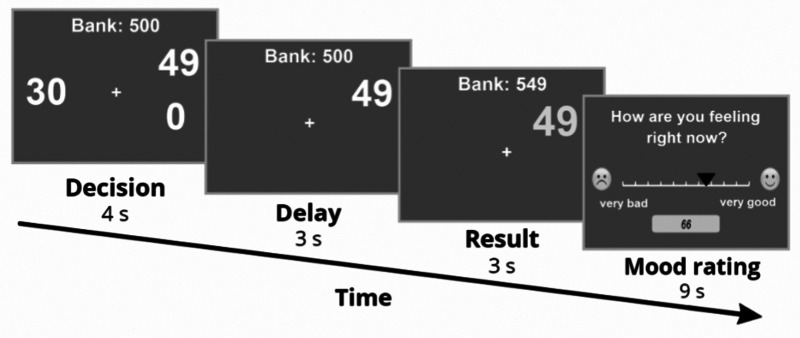
The risky decision-making task with an assessment of momentary mood. Participants indicated their decision with a button press and reported on their momentary mood by clicking on the scale with a mouse. For a detailed description of the task and the computational model, please see the text (section 2.2).

We modeled our experimental paradigm after the one implemented by Rutledge et al. (2014). The study participants filled out an assortment of questionnaires (Beck Depression Scale, Schema Mode Inventory, demographic questions). Apart from this, they took part in a probabilistic decision-making task presented to them as a computer game, where they needed to increase their initial 500 points by as many as possible. In the game, participants made choices between certain and risky monetary options. Furthermore, participants were asked to report their momentary happiness on a scale of 1 (very unhappy) to 100 (very happy) every two to three decisions (Fig. 1). The value of the certain and risky options varied – with the certain option's value always being between the winning and losing conditions' values of the risky option – and participants was presented with 60 trials of winning (the gamble's losing condition was 0), 60 trials of losing (the gamble's winning condition was 0), and 30 trials of mixed (the gamble's losing outcome was negative, while the winning condition was positive and the certain option was 0) outcomes sorted into five blocks, separated by breaks of discretionary length. They were correctly informed that their chances of winning were 50% upon choosing the risky option and that each time they had 9 s to rank their current mood, while only 4 to decide between the certain and risky monetary options, lest they wished to end up with the most unfavorable outcome automatically.

We used the Computational Model of Momentary Subjective Well-being to process the collected data. This model defines momentary happiness in the following way:
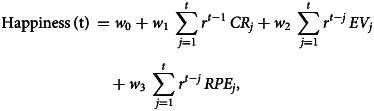
Where *t* is the trial number, while *j* indicates the number of the currently examined trial, *γ* is the forgetting factor, which ensures recent outcomes have a bigger impact in predicting momentary happiness. w0 marks a constant value of base mood, whereas w1, w2, and w3 represent the influence of their corresponding events. The value of Certain Reward (CR) only has predicting power if, on the given trial, the certain reward had been selected. Otherwise, w1 takes up the value of 0 and only gamble Expected Value (EV, which is the average of the possible gamble outcomes), and Reward Prediction Error (RPE, which is the difference between realized and expected rewards) affects momentary happiness.

### SCl-90, Beck depression inventory, and Young Schema Mode Inventory (YSI)

The 124-item Young Schema Mode Inventory assesses the frequency of 14 schema modes' activation (Lobbestael et al., 2010; Young et al., 2007). The model consists of five innate child modes, five dysfunctional coping modes, two dysfunctional parent modes, and the adaptive Healthy Adult mode (See Table 1). Cronbach's *α* coefficients of the schema mode subscales in this study ranged from (0.62) to (0.92). Answers are rated on a 6-point Likert scale (from 1 – ‘Never’ or ‘Almost Never’ to 6 – ‘All of the time’). The Hungarian adaptation of the YSI was applied in previous studies (Salgó et al., 2021; Szalai, 2014).

The *Symptom Checklist-90* [SCL-90, Derogatis et al. (1973); the Hungarian version (Unoka *et al*., 2004) is a widely used 90-item scale for assessing self-reported psychological distress and psychopathology. The SCL-90 measures a broad range of psychological problems and symptoms of psychopathology, which are related to Somatization, Obsessive-Compulsive, Interpersonal Sensitivity, Depressive, Anxiety, Hostility, Phobic Anxiety, Paranoid Ideation, and Psychoticism. Each item of the questionnaire is rated on a five-point distress scale from 0 (none) to 4 (extreme).

### Statistical analysis

#### The solution of the computational model: Hierarchical Bayesian Analysis (HBA)

To estimate model parameters, we used the ‘hBayesDM Package’ developed by Ahn et al. (Ahn, Haines, & Zhang, 2017), which implements a hierarchical Bayesian analysis (HBA). In Bayesian data analysis, Bayes' rule is used to update priors (assumed starting values of model parameters) using measured data to estimate posterior distributions (Kruschke, 2015a). Compared to the more conventional Maximum Likelihood Estimation, the advantage of this approach is that it estimates both individual and group parameters (i.e. posterior distributions) simultaneously in a mutually constraining fashion. Consequently, individual parameter estimates tend to be more stable and reliable since commonalities among individuals are captured and informed by the group tendencies (Ahn, Krawitz, Kim, Busmeyer, & Brown, 2011). HBA also finds full posterior distributions instead of point estimates that can be used in (Bayesian fashion) group comparisons. The Hamiltonian Monte Carlo (HMC) algorithm was used [a Markov Chain Monte Carlo algorithm implemented in Stan software package: [https://mc-stan.org/; (Kruschke, 2015c)] to find model parameter estimates (posterior distributions). According to the recommendation of Ahn et al. (Ahn, Haines, & Zhang, 2017) and Valton, Wise, and Robinson (2020), we estimated the model parameters for the two groups separately as this way parameter estimation tends to lead to more accurately recovered group differences. The Bayesian Inference (‘null hypothesis’) was used to compare model parameters between study groups. The difference between model parameter distributions of study groups was calculated for each parameter separately, what was considered significant if zero was outside of the 95% High Density Interval (HDI) of the calculated difference. The 95% HDI is the credible interval within which the estimated (i.e. unobserved) parameter value falls with 95% probability (Kruschke, 2015b). Raw task data that can be used to reproduce the models is available here: https://osf.io/vxwnr/.

#### Schema modes, depression severity, and task performance

Depression severity, schema modes, and task performance were compared by the Mann–Whitney *U* test between study groups, and effect sizes were presented in Cliff's Delta. Model parameters were compared by the Wilcoxon non-parametric test between medication-free and medication-taking patients. This latter analysis was performed separately for the different types of psychotropic medications such as APs, ADs, MSs, and BZDs.

#### Correlational analyses

The relationship of model parameters with schema modes, BZD dose (in terms of diazepam equivalents), and AP dose (in terms of Chlorpromazine equivalents) was investigated with Spearman-correlations. In the case of schema modes, the Holm–Bonferroni method was used to correct for multiple testing. First analyses were performed for all participants, and a correction was applied. Where a significant result was detected, correlations were analyzed within each study group separately.

## Results

### Task performance, schema modes, and depressive symptoms

For descriptive purposes, we present the comparison of patients and controls in terms of depressive symptoms, schema modes, and simple indicators of task performance such as the proportion of trials where they decided to gamble, total money earned in the task, and reaction time of decisions and happiness ratings (Table 3). Unsurprisingly, we observed higher levels of depressive symptoms and a more maladaptive schema mode profile among patients with BPD. There was notable heterogeneity within the patient group regarding depression and schema modes, which we will address in greater detail in section 3.5. Furthermore, patients with BPD earned less than controls [79.5 (s.d. = 426) *v.* 322 (s.d. = 467), Mann–Whitney *p* = 0.03, Δ = −0.32 (−0.56 to −0.03)]. There were no significant differences between the two groups in terms of proportion of gambling [57.8% (s.d. = 17.1) *v.* 54.7% (s.d. = 18.1), Mann–Whitney *p* = 0.747, Δ = 0.05 (−0.24 to −0.33)] or reaction times [mood decision RT: 3.7 (s.d. = 0.9) *v.* 3.5 (s.d. = 0.6), Mann–Whitney *p* = 0.717, Δ = 0.05 (− 0.24 to −0.34); monetary decision RT: 1.6s (s.d. = 0.4) *v.* 1.7s (s.d. = 0.3), Mann–Whitney *p* = 0.79, Δ = −0.04 (−0.32 to −0.25)].

**Table 3.** Descriptive statistics for depressive symptoms and schema modes

Variable	Borderline personality disorder		Healthy controls		Cliff's delta [95%CI]	Mann–Whitney *p* value
	Mean (s.d.)	Cronbach's Alpha	Mean (s.d.)	Cronbach's Alpha		
Depressive symptoms (BDI)	24.6 (11.7)	0.90	3.4 (3.8)	0.76	0.93 [0.76–0.98]	<0.001
Vulnerable child	3.9 (1.2)	0.92	1.7 (0.7)	0.90	0.83 [0.58–0.94]	<0.001
Angry child	3.2 (1.1)	0.85	1.8 (0.5)	0.72	0.77 [0.52–0.9]	<0.001
Enraged child	2 (0.9)	0.89	1.1 (0.2)	0.66	0.61 [0.34–0.78]	<0.001
Impulsive child	2.8 (0.9)	0.85	2 (0.5)	0.68	0.52 [0.23–0.72]	0.001
Undisciplined child	3.4 (1)	0.70	2.3 (0.7)	0.49	0.67 [0.4–0.83]	<0.001
Compliant surrender	3.4 (1.1)	0.84	2.7 (0.8)	0.56	0.36 [0.06–0.6]	0.015
Detached protector	3 (1.1)	0.89	1.6 (0.6)	0.82	0.71 [0.47–0.85]	<0.001
Detached selfsoother	4.3 (1)	0.59	2.4 (0.9)	0.72	0.8 [0.57–0.91]	<0.001
Self-aggrandizer	2.9 (1)	0.86	2.4 (0.7)	0.79	0.29 [0–0.54]	0.05
Bully	2.1 (0.7)	0.71	1.5 (0.3)	0.22	0.54 [0.26–0.74]	<0.001
Punitive parent	3.4 (1.3)	0.92	1.4 (0.4)	0.75	0.84 [0.64–0.93]	<0.001
Demanding parent	4.2 (0.9)	0.77	3.2 (0.8)	0.78	0.63 [0.37–0.8]	<0.001
Healthy adult	3.6 (0.9)	0.80	4.6 (0.5)	0.59	−0.64 [−0.81 to −0.38]	<0.001

### Model diagnostics

The model was estimated with 3000 iterations (plus 1000 warmup iterations not used for analysis) and six chains resulting in 18 000 estimations per parameter per subject. Vehtari, Gelman, Simpson, Carpenter, and Bürkner (2021) suggest a minimum of four chains and 1000 iterations. Additionally, the convergence of the MCMC algorithm was monitored by the *Ř* value. The sampling can be used if the *Ř* value is less than 1.05 (Vehtari et al., 2021), while the maximum value of *Ř* in the present analysis was 1.0036 in both study groups combined [Detailed trace plots of the MCMC algorithm are in online Supplementary Fig. S1]. The model explained trial-to-trial fluctuations in the mood with an r square of 0.57 (Total sample: s.d. = 0.12, min = 0.35, max = 0.97). Explained variance was similar in both study groups [HC *r* square = 0.61 (s.d. = 0.12); BPD r square = 0.54 (s.d. = 0.11) [Explained variance by subject in online Supplementary Fig. S2].

### Difference between study groups in model parameters: w0, w1, w2, and w3

**Fig. 2.**
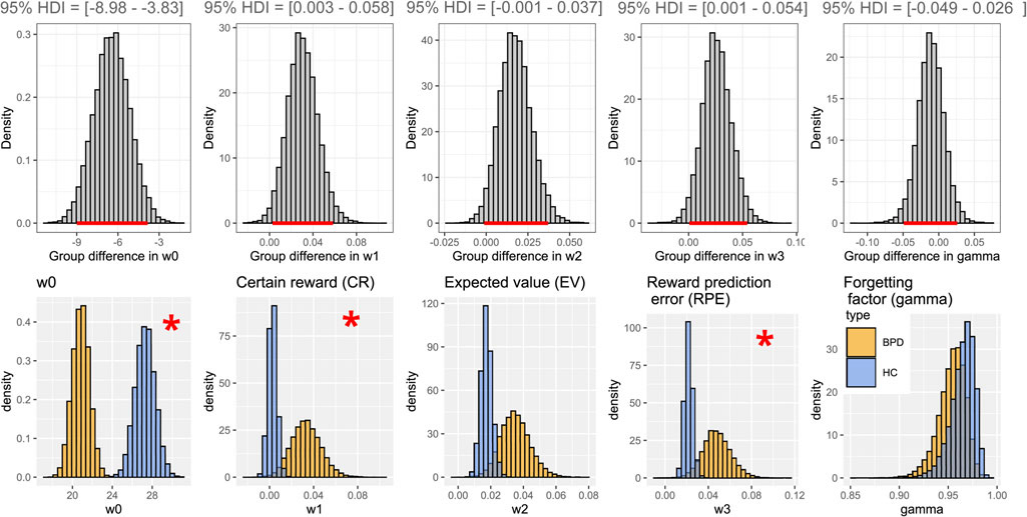
Upper panel: Difference between model parameter distributions of study groups. Horizontal red lines on the x-axes mark 95% credible intervals. Bottom panel: Model parameter distributions for study groups. Healthy controls (HC): orange; Borderline personality disorder (BPD): blue Asterisks mark significant differences between study groups for the given model parameter.

All five model parameters were positive in both study groups. In patients with BPD (BPD), the w0 parameter was lower compared to healthy controls [w0: 95% HDI of the difference = (−8.98 to −3.83)], while the effect of certain rewards [CR: 95% HDI of the difference = (0.003–0.058)] and reward prediction errors (RPE = the difference between expected and experienced rewards) on mood were higher [RPE: 95% HDI of the difference = (0.001–0.054)]. There was no between-group difference in the effect of expected rewards of gambles [EV: 95% HDI of the difference = (−0.001 to 0.037)], and in the effect of forgetting factor [gamma: 95% HDI of the difference = (−0.049 to 0.026)] (Fig. 2). However, the between-group difference in expected rewards of gambles can be considered a statistical trend.

### Correlations of model parameters with schema modes

**Fig. 3.**
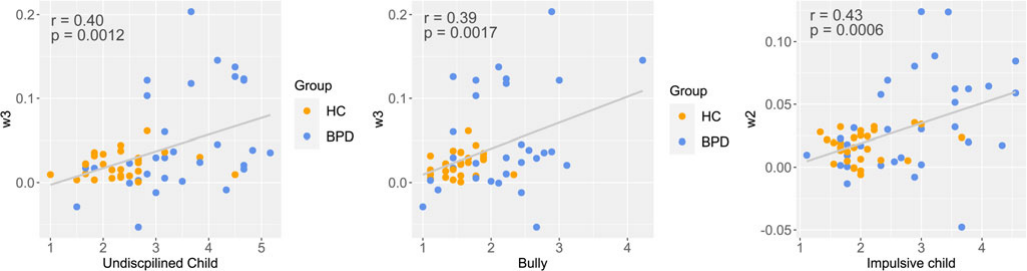
Correlations between schema modes and model parameters. Since model parameters do not fulfill the criteria of normality to apply parametric statistical methods, the Spearman correlation was used. Therefore, regression lines were presented only for visualization purposes.

As we noted above, there was remarkable heterogeneity within the patient group. Therefore, we analyzed the relationship between schema modes and model parameters characterizing mood and its responsivity to outcomes during the gambling task by Spearman correlations. The 95% Confidence Intervals for Spearman Rhos are presented in square brackets. After correcting for multiple testing, the correlation of w2 and the ‘Impulsive Child’ schema mode [All subjects: Spearman *r* = 0.43 (0.19–0.61), *n* = 61, *p* = 0.0006; BPD group: Spearman *r* = 0.45 (0.12–0.69), *n* = 32, *p* = 0.0094; Control group: Spearman *r* = 0.23 (−0.16 to 0.54), *n* = 29, *p* = 0.24] and the correlation of w3 and the ‘Undisciplined Child’ [Spearman *r* = 0.40 (0.17–0.59), *n* = 61, *p* = 0.0012; BPD group: Spearman *r* = 0.48 (0.15–0.71), *n* = 32, *p* = 0.0054; Control group: Spearman *r* = 0.20 (−0.18 to 0.52), *n* = 29, *p* = 0.30] and ‘Bully Attack’ [Spearman *r* = 0.39 (0.15–0.58), *n* = 61, *p* = 0.0017; BPD group: Spearman *r* = 0.34 (−0.01 to 0.61), *n* = 61, *p* = 0.06; Control group: Spearman *r* = 0.41 (0.05–0.67), *n* = 29, *p* = 0.03] schema modes remained significant (Fig. 3). The w0 parameter correlated negatively with all the schema modes (Spearman rhos were between −0.46 and −0.78), except the ‘Happy Child’ and the ‘Healthy Adult’ traits, where the correlations were positive (Spearman *r* were between 0.54 and 0.66). The w1 and gamma parameters did not correlate significantly with any of the schema modes after correction.

In order to evaluate the specificity of the effects, we performed a principal components analysis using *z* standardized schema mode scores (for details see online Supplementary Materials). The first principal component explained around 60% of the variance, while the second explained about 10%. The scree plot clearly suggested retaining the first principal component (online Supplementary Fig. S3.2). Retaining only the first component was confirmed by inspection of the loadings (online Supplementary Fig. S3.3), which were straightforward to interpret for the first component: all maladaptive schema modes demonstrated a negative loading, and adaptive schema modes (e.g. ‘healthy adult’, ‘happy child’) had a positive loading. Therefore, we decided to use the first principal component in further analyses. For the sake of interpretability, we reversed the scores, so that higher scores represent more maladaptive schemas.

We evaluated the association of maladaptive schemas with model parameters in a series of multiple linear regression models. In general, the pattern of results aligned well with the scale-level findings in that depression and maladaptive schemas were both related to w0, while w1, w2, and w3 were specifically associated with the level of maladaptive schemas (online Supplementary Fig. S3.5 and Table S1).

### Correlations of model parameters with age, education, and Beck depression score

Age and education did not correlate with any of the model parameters. Beck depression score correlated negatively with the w0 model parameter (Spearman *r* = −0.77, *n* = 61, *p* < 0.0001; BPD group: Spearman *r* = −0.74, *n* = 32, *p* < 0.0001; Control group: ns.), but not with the other model parameters.

### Relationship of model parameters with medications

Model parameters (w0, w1, w2, and w3) did not differ (Wilcoxon non-parametric test *p* > 0.05 after correction for multiple comparisons) between patients using antipsychotic medication, benzodiazepines, mood stabilizers, and antidepressants *v.* those patients who did not. The only exception was the effect of antidepressant use on the forgetting factor (gamma), where patients on AD treatment had a significantly lower gamma value [AD *v.* non-AD: 0.87 (s.d. = 0.07) *v.* 0.94 (s.d. = 0.08), Wilcoxon *Z* = 2.9, *p* = 0.007]. Also, antipsychotic dose in terms of chlorpromazine equivalents and benzodiazepine dose in terms of diazepam equivalents did not correlate significantly (Spearman correlation *p* > 0.05) with model parameters (w0, w1, w2, and w3).

## Discussion

We aimed to establish how affective instability is influenced by reward expectation, outcomes, and RPEs during risky decision making in patients with BPD compared to non-clinical controls. Moreover, we adopted a dimensional approach and looked at associations with maladaptive and adaptive schema modes, which capture variation in BPD-like information processing and emotion regulation alterations. We used an established paradigm of mood fluctuations during risky decision-making, which explicitly quantifies the extent to which expected and realized outcomes, and RPEs influence momentary mood. To our knowledge, this is the first study investigating mood fluctuations of patients with BPD during decision making. The computational model in this study informs us how expectations and surprises connected to expected rewards influence our momentary well-being in a decision-making situation. All model weights were found to be positive, which means that positive expectations regarding certain rewards and gambles and positive surprises had a positive effect on mood, while negative expectations and negative surprises had an opposite effect. The applied model explained fluctuations in mood well in both study groups (see online Supplementary Fig. S2 for details) and explained variance in terms of r square was comparable to that reported previously by Rutledge et al. (2014). Our major finding is that w3, the weight for RPE, is higher in patients with BPD; in other words, the difference between expectations and outcomes (i.e. surprise) has a greater impact on BPD patients' mood as compared to what we observed in healthy participants. This finding may give a deeper look into the abrupt mood shifts experienced by patients.

Modeled baseline mood (w0) was lower in patients, which is well in line with the higher level of depressive symptoms among patients. This notion is also backed by the strong correlation of w0 with depression severity measured by the Beck Depression Inventory and with all schema modes. Drawing on the rich literature on affective instability in BPD, we expected that the mood of patients with BPD would be more responsive to outcomes, expectations, and their mismatch during risky decision-making. According to our expectations we found that the mood of BPD patients was more responsive to the magnitude of certain rewards and RPEs: the w1 weight of certain rewards (CR) and the w3 weight of RPEs were significantly higher in patients with BPD compared to controls. Additionally, the w2 effect of expected rewards in gambles (EV) showed a similar trend. Based on these findings, patients with BPD seem to respond to changes in the game with stronger mood fluctuations. Furthermore, the effect of expected rewards in gambles (EV) and RPE increased with maladaptive schema modes such as ‘Impulsive child’ and ‘Undisciplined child’ showing that the mood of patients with stronger maladaptive schema modes can be modulated easier. The fact that one general factor comprised all the schemas (maladaptive and adaptive one with opposite sign) possibly mean that higher maladaptive schema scores indicate more severe borderline psychopathology, which notion is also in line with previous findings (Sharp et al., 2015). A correlation was found between this general factor and the model parameters showing a possible positive relationship between the severity of borderline symptomatology and the impact of model parameters on mood. As per Young et al. (2006), when these maladaptive schema modes are active in individuals, they ‘act on non-core desires or impulses in a selfish or uncontrolled manner to get their way and often have difficulty delaying short-term gratification, they often feel intensely angry, enraged, infuriated, frustrated, impatient when these non-core desires or impulses cannot be met.’ This phenomenological description strikingly well explains the mood shifts during the game. Suppose the applied paradigm is considered a model framework for positive and negative life events and decisions. In that case, we can interpret our findings as an ‘over-reactivity’ to events in BPD, that may increase with borderline symptom severity. While the frequency of risk-taking behavior was similar in both groups, the effect of their decisions and the decision's outcomes on mood were stronger in patients as compared to controls. Our findings may explain BPD patients' abrupt mood changes, often leading to unpredicted or self-harming behavior.

Furthermore, we found that variation in the impact of reward processing on mood will be related to schema-modes, that is, information processing and self-regulation styles rooted in early relational experiences. Performing analyses from a dimensional perspective are following state-of-the-art dimensional approaches to psychopathology, such as the Research Domain Criteria (Cuthbert, 2020; Sanislow, 2020), or the Hierarchical Taxonomy of Psychopathology (Kotov et al., 2021; Michelini et al., 2021), that contrasts the dimensional approach followed in DSM-5 and ICD-10 (Borsboom et al., 2016). Our dimensional analyses yielded further evidence that we found BPD-specific alterations: the model parameters that capture how rewards impact mood (w1, w2, and w3) and differ between patients with BPD and controls seem to correlate more with schema modes, but not with depressive symptoms. In contrast, a parameter capturing an overall mood level (w0) was strongly associated with depressive symptoms and schema modes, replicating previous findings (Rutledge et al., 2017). Such demonstration of specificity is particularly valuable because patients with BPD show elevated depressive symptoms, which are also highly variable across patients (Köhling, Ehrenthal, Levy, Schauenburg, & Dinger, 2015). This finding aligns with a previous study involving a large sample of patients with major depressive disorder (MDD) using the same task. They reported that patients with MDD did not differ significantly from controls in terms of the emotional impact of gamble expected value or reward prediction error, while in a large general population sample, the severity of depressive symptoms weakly correlated with the greater emotional impact of reward prediction error (Rutledge et al., 2017). Our findings, combined with the results of this previous study with MDD patients, indicate that impairments in reward prediction error may explain mood changes better in BPD than in MDD. Our results also point to the phenomenon of ‘equifinality’ that in MD and BPD, there may be different mechanisms underlying depressive symptoms measured by Beck's Depression Scale. Previous studies found essential similarities and differences between affective psychopathology and its neurobiological underpinnings in MD and BPD (Goodman, New, Triebwasser, Collins, & Siever, 2010). The paradigm we used in this study may help to discern differences in the neurobiology of these two disorders.

The forgetting factor (gamma) did not differ between study groups indicating that events in earlier trials influenced well-being similarly in both study groups. Gamma was higher in this study (gamma ~ 0.9) compared to the previous investigation (gamma ~ 0.6) involving only healthy volunteers (Rutledge et al., 2014), which indicates that earlier events influenced subjects' mood in our experiment more. A Possible reason for the difference is that the previous study was run as a smartphone application and in an MRI, while the present investigation was run in an experimental room.

While abrupt and frequent mood shifts in BPD are prominent features of the disorder, our knowledge of the underlying neurocognitive mechanisms driving these changes and their neurobiology is incomplete. Previous studies with healthy subjects show a direct connection between RPEs and striatal dopamine activity (Schultz, Dayan, & Montague, 1997). A previous fMRI study using the very same probabilistic monetary reward task in healthy volunteers showed that model weights of EV and RPE correlated with BOLD activity in the striatum (Rutledge et al., 2014). In this context, higher EV and RPE weights in the patient group may indicate a striatal over-reactivity in patients with BPD. This notion is in line with recent fMRI findings in BPD patients showing striatal hyperactivity during acceptance of negative emotional stimuli (Lamers et al., 2019). Also, this may indicate a striatal over reactivity in subjects with stronger maladaptive schema modes connected to impulsivity. However, investigations in BPD applying the same paradigm in fMRI are needed to confirm this notion.

There are some limitations of the study. First, most of our participants were females, which decreases the generalizability of our findings. However, the predominance of females fits data on the prevalence of BPD (>70% female) by sex (Lieb et al., 2004). Second, although the correlational analyses yielded theoretically meaningful and robust effects specific to BPD-related schema modes, our study might not be optimally powered for such dimensional analyses, and there is probably an increased risk of false negatives. Future studies should replicate the dimensional findings in large population-based samples where a multitude of psychopathology/mental health dimensions are assessed (Gillan & Rutledge, 2021). A key strength of our study is that we adopted a well-validated formal model of momentary mood, developed by Rutledge et al. This model has been validated against numerous competing alternatives using data from clinical and non-clinical samples that were tested in various settings (lab/scanner/smartphones) (Gillan & Rutledge, 2021). We estimated parameters for this well-validated model using the probabilistic programming language Stan via the Rstan and the hBayesDM interfaces. For transparency and reproducibility, we share the raw data (https://osf.io/vxwnr/); this way, interested readers may evaluate novel alternative models. Finally, a significant limitation of the present study is that several patients took psychotropic medications. However, we did not find differences in model parameters between medicated and unmedicated patients, and there were no correlations between model parameters and medication dose.

## Conclusions

Our findings show that BPD is coupled with altered associations between mood fluctuation and reward processing under uncertainty. One is tempted to speculate that these may lead to the core symptoms of BPD, such as mood swings, impulsivity, and pervasive instability of interpersonal relationships. Should that be the case, our study has pointed toward a core information processing alteration that may serve as a risk detection measure and could be targeted with specific psychological and biological therapies.

## Supplementary material

For supplementary material accompanying this paper visit https://doi.org/10.1017/S0033291723000193. click here to view supplementary material
