# Supplementary material for: Stronger coupling of emotional instability with reward processing in borderline personality disorder is predicted by schema modes
Source: Psychol Med. 2023 Feb 9;53(14):6714–23. doi: 10.1017/S0033291723000193 (PMC10600820; doi:10.1017/S0033291723000193)
Supplement: Supplementary file 1 [file S0033291723000193sup001.pdf]

Supplement

Stronger coupling of emotional instability with reward processing in borderline personality disorder is predicted by schema modes

1. Detailed trace plots of the MCMC algorithm by group

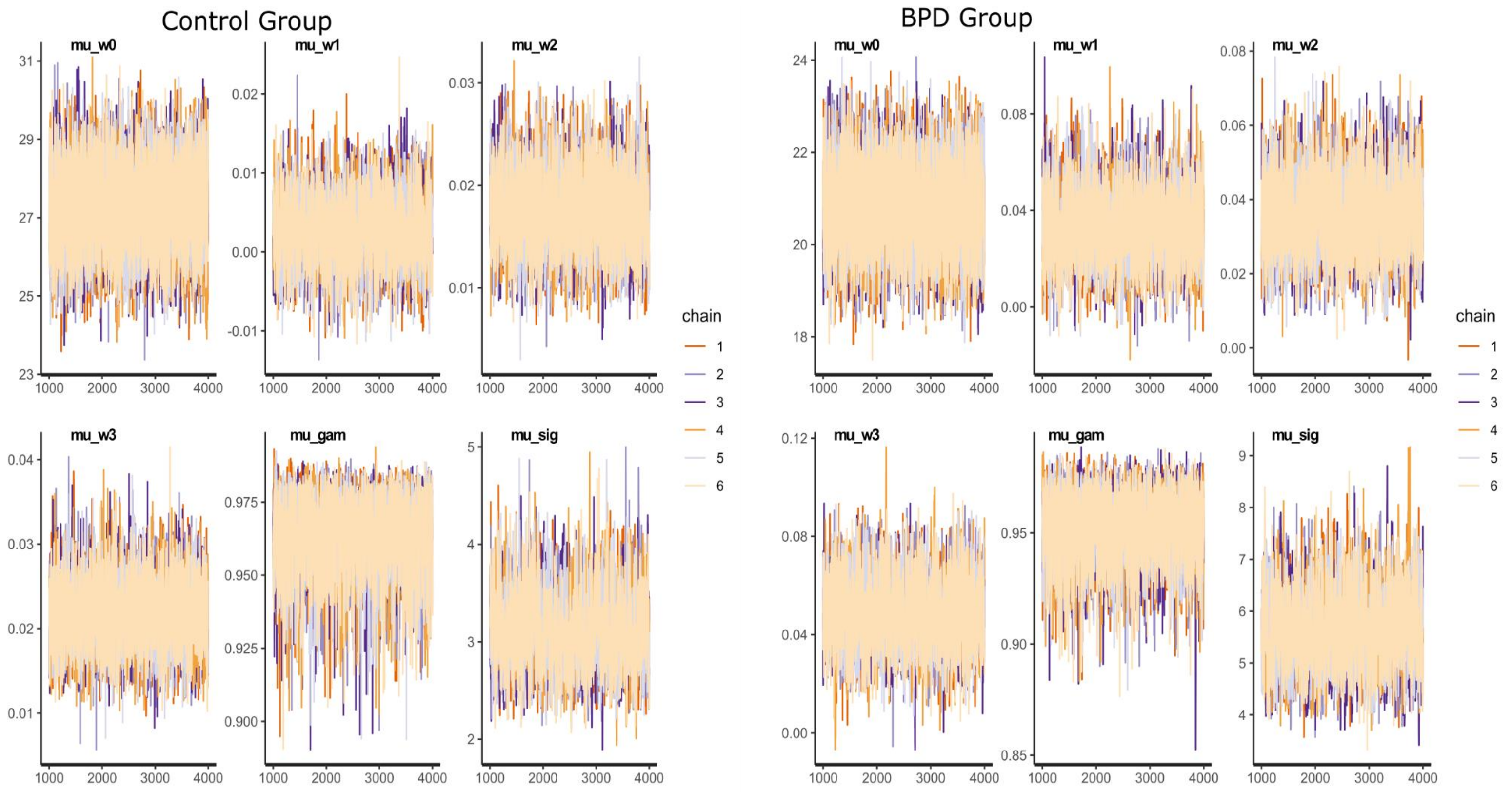

2. Estimated (predicted) mood vs. assessed (actual) mood by subject

a.) Control group (actual mood ; predicted mood from the model)

1

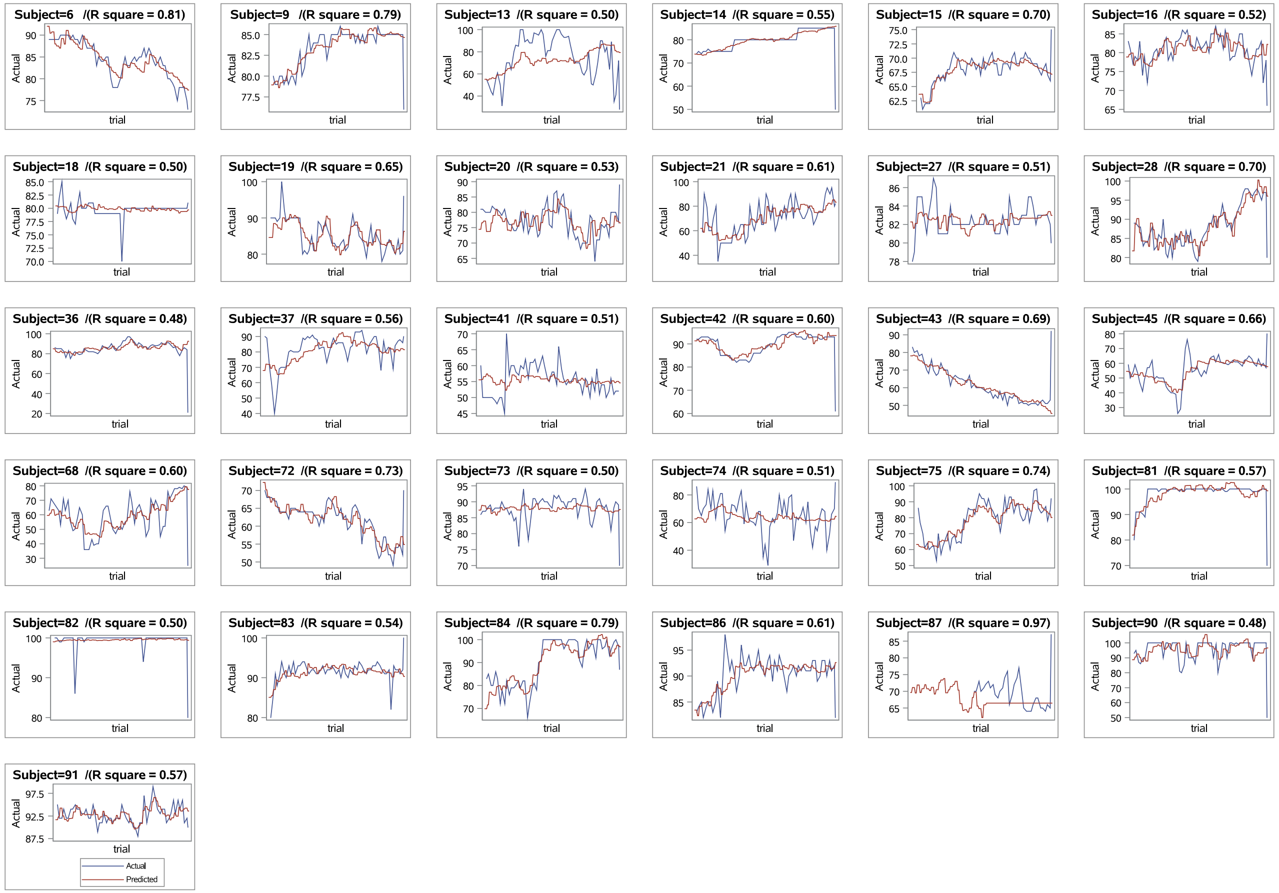

b.) BPD group (actual mood ▬ ; predicted mood from the model ▬)

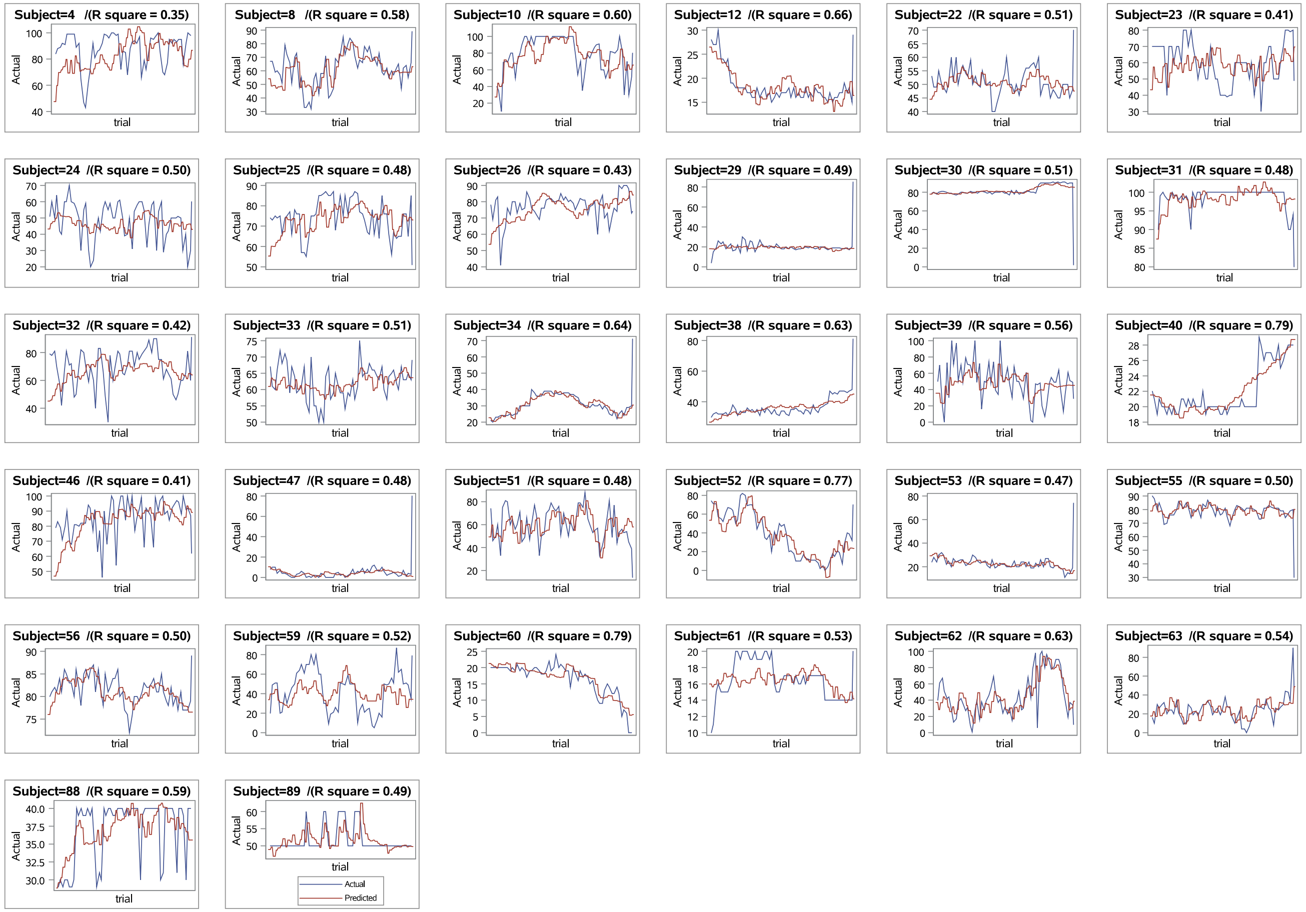

3. General schema mode maladaptation as a predictor of model parameters

First, we examined the correlations of schema mode subscales (Supplementary Figure 3.1). The subscales showed strong intercorrelations, so it was not feasible to enter them together into a regression model (due to multicollinearity). To evaluate the specificity of the effects, we performed a principal components analysis using z-standardized schema mode scores. The first principal component explained around 60% of the variance, while the second explained about 10%. The scree plot clearly suggested retaining the first principal component (Supplementary Figure 3.2).

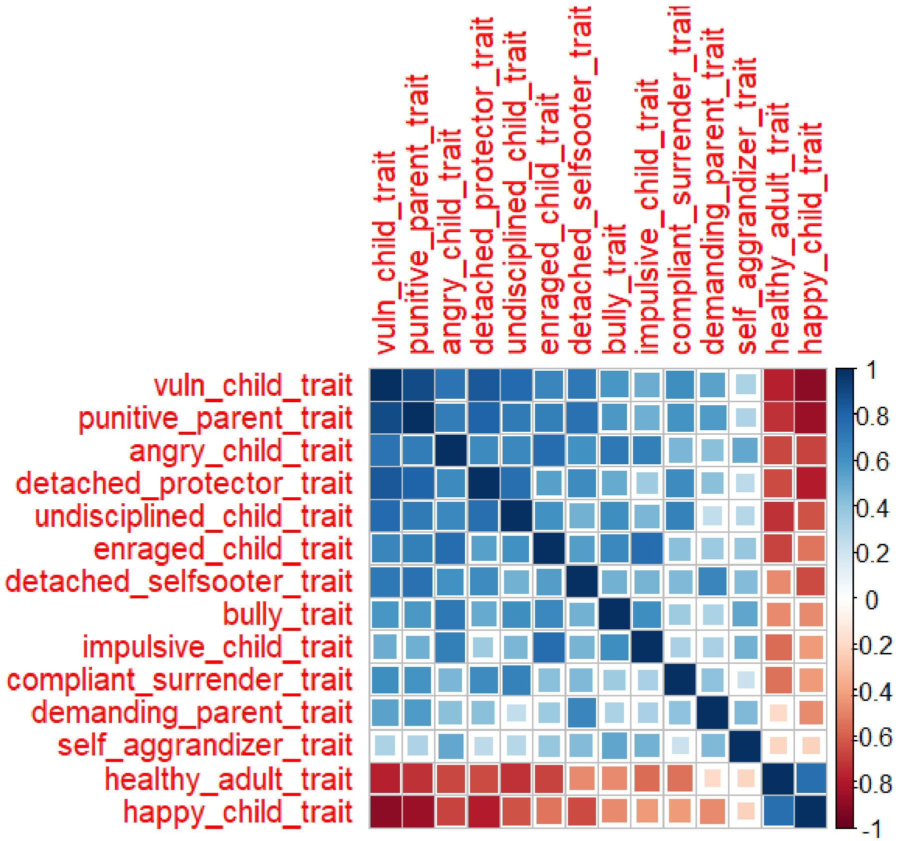

Supplementary Figure 3.1. Association of Schema Mode Inventory subscale scores in the whole sample. The hue reflects Pearson correlation coefficients. Variables are sorted by their loading on the first principal component, in descending order.

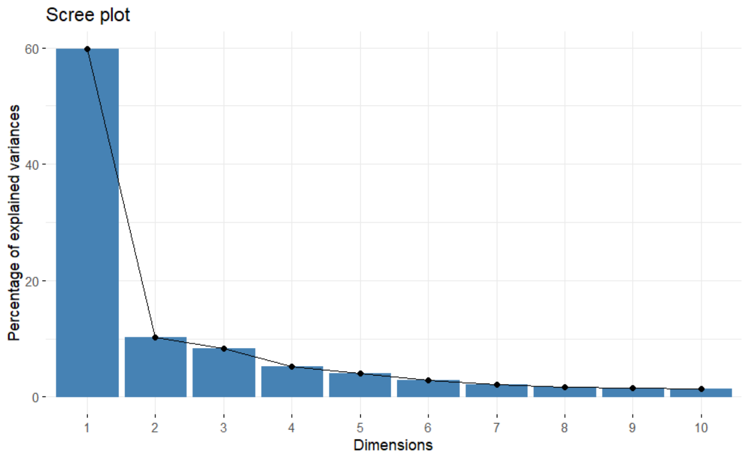

Supplementary Figure 3.2. Scree plot showing the % of variance explained by principal components extracted from the Schema Mode Inventory subscale scores in the whole sample. The scores were z-standardized prior to performing the principal components analysis.

Retaining only the first component was confirmed by inspection of the loadings (Supplementary Figure 3.3), which were straightforward to interpret for the first component: all maladaptive schema modes demonstrated a negative loading, and adaptive schema modes (e.g. 'healthy adult', 'happy child') had a positive loading. Also, this principal component clearly discriminated between patients and healthy controls. Critically, the second component was not clearly interpretable and did not differentiate between the two groups. Therefore, we decided to use the first principal component in further analyses. For the sake of interpretability, we reversed the scores, so that higher scores represent more maladaptive schemas.

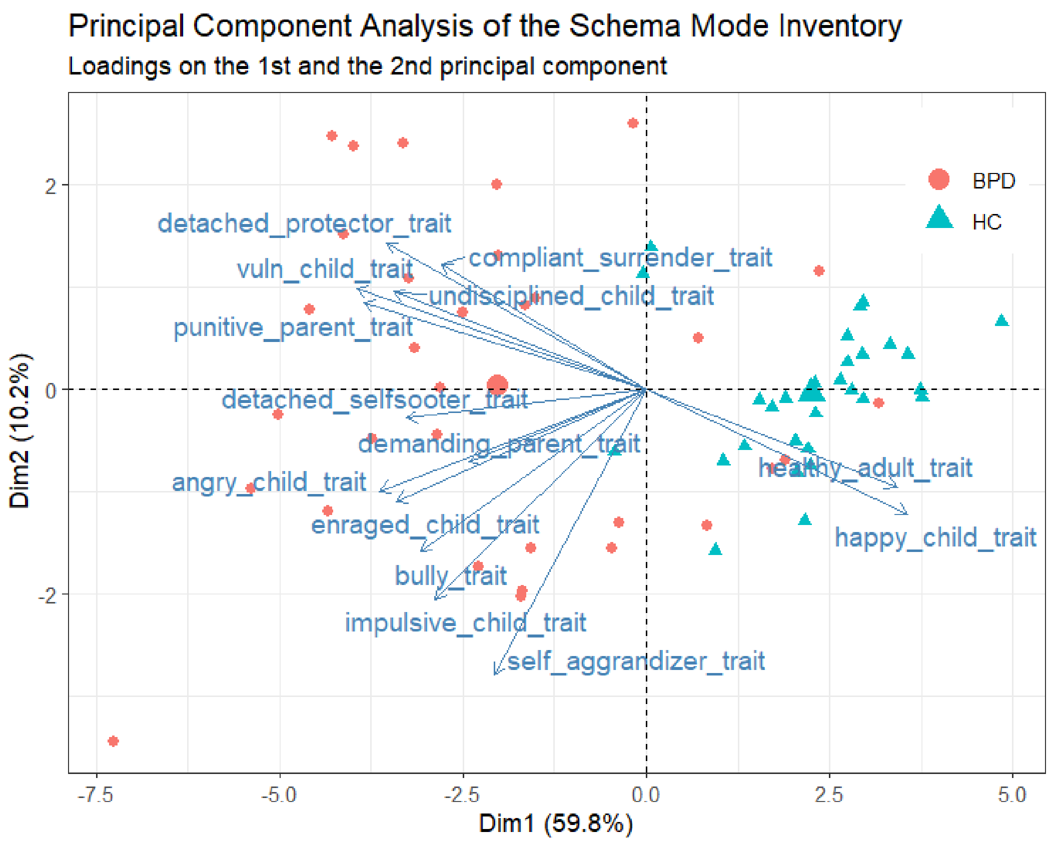

Supplementary Figure 3.3. Biplot showing loadings of Schema Mode Inventory subscale scores on the first and the second principal component on the x and the y axis, respectively. Individual participants are plotted in a two-dimensional space obtained with a Principal Component Analysis.

The severity of maladaptive schema modes (as reflected by the first principal component) was positively related to the severity of depressive symptoms (as indicated by the Beck Depression Inventory total score, Supplementary Figure 3.4). As our aim was to establish that maladaptive schemas are specifically associated with model parameters over and above the effect of depression, we entered the depression score into every regression model.

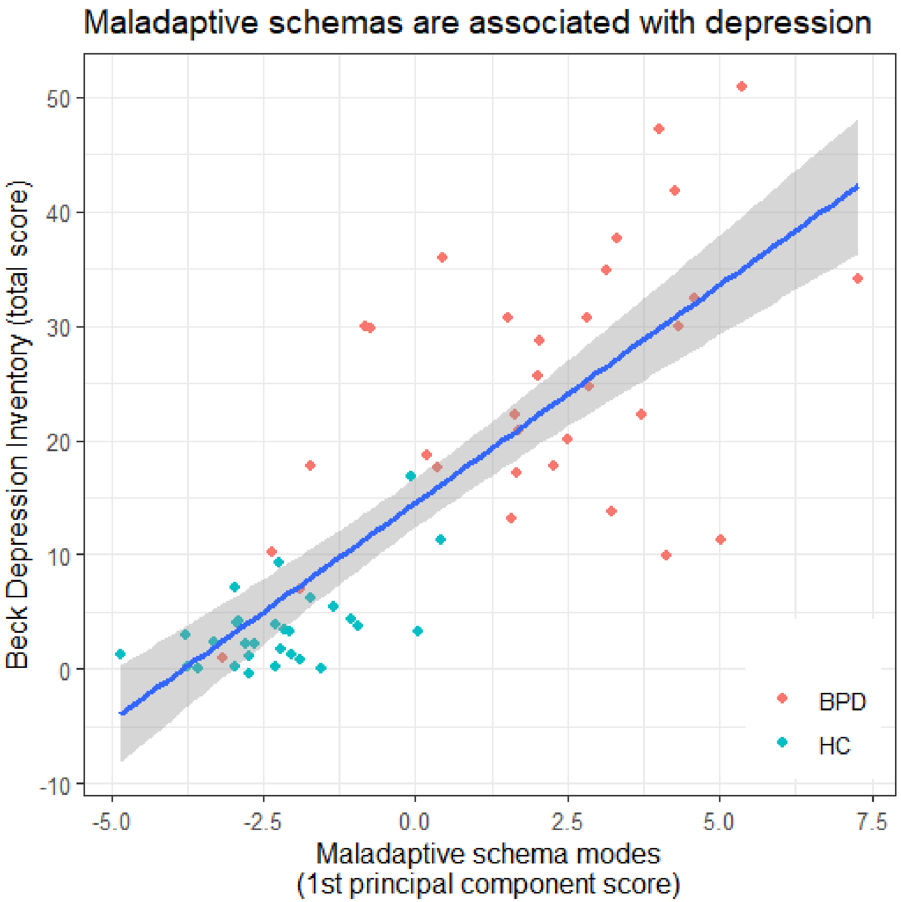

Supplementary Figure 3.4. The association of maladaptive schema modes and severity of depressive symptoms. The line indicates linear fit, and the shading shows the 95% confidence interval.

We evaluated the association of maladaptive schemas with model parameters in a series of multiple linear regression models. Maladaptive schemas and depression severity were the predictors, and model parameters (w0, w1, w2, w3 and gamma) were the dependent variables. Standardized coefficients from the models are shown in Supplementary Figure 3.5, followed by a summary in Supplementary Table 1. In general, the pattern of results aligned well with the scale-level findings in that depression and maladaptive schemas were both related to w0, while w1, w2, and w3 were specifically associated with the level of maladaptive schemas.

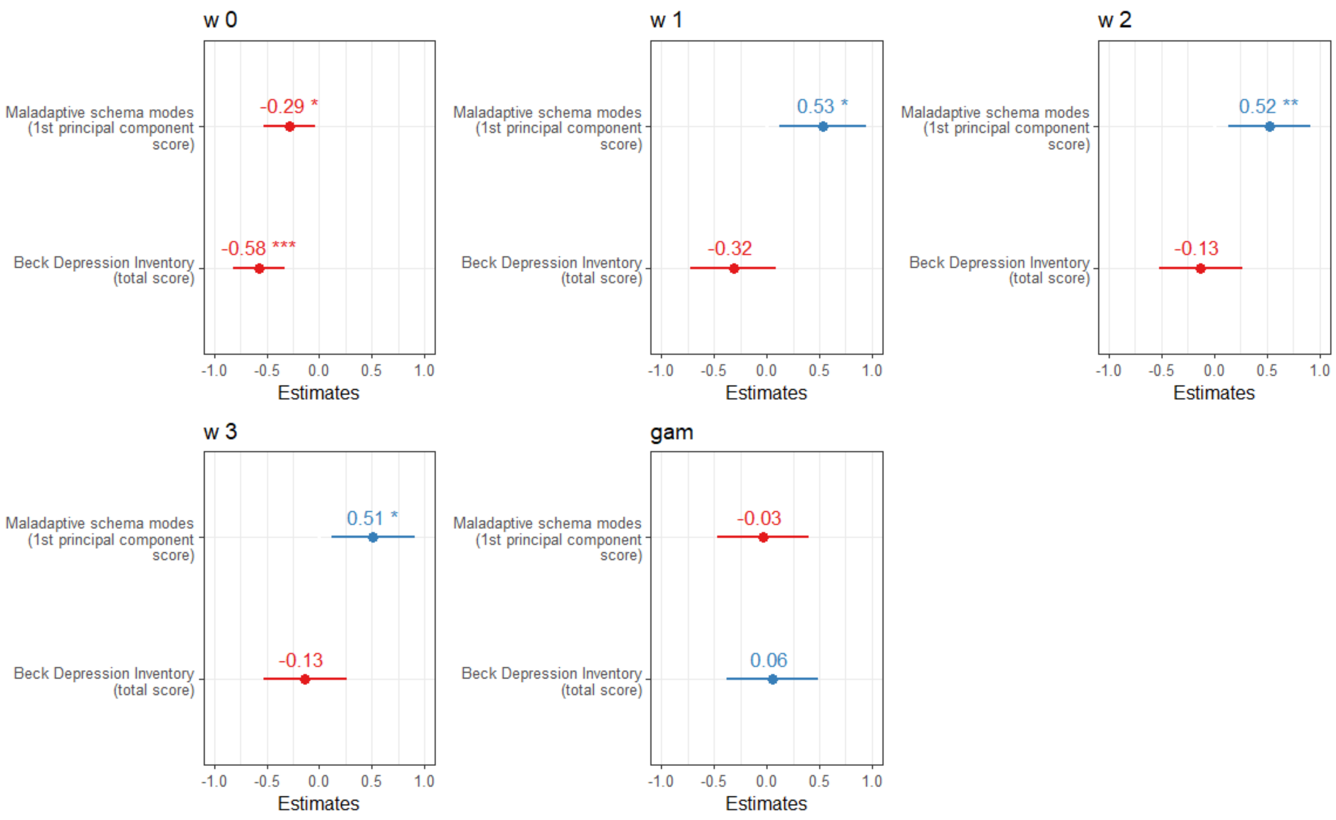

Supplementary Figure 3.5. A visual summary of linear regressions predicting model parameters from maladaptive schemas and depressive symptoms. Standardized beta coefficients and their 95% confidence intervals are shown. gam=forgetting factor gamma

Predictors	w0 *std. Beta*	w0 *standardized CI*	w0 *p*	w1 *std. Beta*	w1 *standardized CI*	w1 *p*	w2 *std. Beta*	w2 *standardized CI*	w2 *p*
(Intercept)	-0.00	-0.15 – 0.15	**<0.001**	0.00	-0.25 – 0.25	**0.005**	0.00	-0.24 – 0.24	**<0.001**
Maladaptive schemas – principal component	-0.29	-0.53 – -0.04	**0.024**	0.53	0.13 – 0.94	**0.011**	0.52	0.13 – 0.92	**0.010**
Beck total score	-0.58	-0.82 – -0.33	**<0.001**	-0.32	-0.72 – 0.09	0.126	-0.13	-0.52 – 0.27	0.522
Observations	61			61			61		
R^2 / R^2 adjusted	0.676 / 0.665			0.116 / 0.086			0.185 / 0.157		

Predictors	w3 *std. Beta*	w3 *standardized CI*	w3 *p*	gamma *std. Beta*	gamma *standardized CI*	gamma *p*
(Intercept)	-0.00	-0.24 – 0.24	**<0.001**	-0.00	-0.26 – 0.26	**<0.001**
Maladaptive schemas – principal component	0.51	0.11 – 0.90	**0.013**	-0.03	-0.46 – 0.40	0.885
Beck total score	-0.13	-0.53 – 0.26	0.503	0.06	-0.38 – 0.49	0.798
Observations	61			61		
R^2 / R^2 adjusted	0.168 / 0.140			0.001 / -0.033		

Supplementary Table 1. Summary of linear regressions predicting model parameters from maladaptive schemas and depressive symptoms. Standardized beta coefficients and their 95% confidence intervals are shown.
